# Variants in microRNA genes in familial papillary thyroid carcinoma

**DOI:** 10.18632/oncotarget.14129

**Published:** 2016-12-23

**Authors:** Jerneja Tomsic, Rebecca Fultz, Sandya Liyanarachchi, Luke K Genutis, Yanqiang Wang, Wei Li, Stefano Volinia, Krystian Jazdzewski, Huiling He, Paul E Wakely, Leigha Senter, Albert de Chapelle la

**Affiliations:** ^1^ Department of Cancer Biology and Genetics, The Ohio State University Wexner Medical Center and Comprehensive Cancer Center, The Ohio State University, Columbus, OH, USA; ^2^ Deptartment of Morphology, Surgery and Experimental Medicine, University of Ferrara, Ferrara, Italy; ^3^ Genomic Medicine, Medical University of Warsaw, Warsaw, Poland; ^4^ Laboratory of Human Cancer Genetics, Centre of New Technologies, CENT, University of Warsaw, Warsaw, Poland; ^5^ Department of Pathology, Arthur G. James Cancer Hospital and Richard J. Solove Research Institute, The Ohio State University Wexner Medical Center, Columbus, OH, USA; ^6^ Department of Internal Medicine, The Ohio State University Wexner Medical Center and Comprehensive Cancer Center, The Ohio State University, Columbus, OH, USA; ^7^ Division of Biomarkers Early Detection Prevention, City of Hope, Duarte, CA, USA

**Keywords:** genetics, predisposition, miRNA, thyroid, variants

## Abstract

Papillary Thyroid Carcinoma (PTC) displays one of the highest familiality scores of all cancers as measured by case-control studies, yet only a handful of genes have been implicated until now. Variants in microRNAs have been associated with the risk of several cancers including PTC but the magnitude of this involvement is unclear. This study was designed to test to what extent genomic variants in microRNAs contribute to PTC risk. We used SOLiD technology to sequence 321 genomic regions encoding 427 miRNAs in one affected individual from each of 80 PTC families. After excluding variants with frequency ≥ 1% in 1000 Genomes Phase 1 (*n* = 1092) we detected 1978 variants. After further functional filtering steps 25 variants in pre-miRs remained. Co-segregation was observed for six out of 16 tested miRNA variants with PTC in the families, namely let-7e, miR-181b, miR-135a, miR-15b, miR-320, and miR-484. Expression of miR-135a and miR-181b was tested in normal thyroid and tumor tissue from patients that carry the variants and a decrease in expression was observed. *In vitro* assays were applied to measure the effect of the variants on microRNAs’ maturation. Four out of six variants were tested. Only the let-7e and miR-181b variants showed an effect on processing leading to lower levels of mature miRNA. These two variants were not detected in 1170 sporadic PTC cases nor in 1404 controls. Taken together, our data show that high penetrance germline sequence variants of miRNAs potentially predispose to a fraction of all PTC but are not common.

## INTRODUCTION

Papillary thyroid cancer (PTC) is an endocrine malignancy that accounts for almost 90% of all thyroid malignancies (SEER Cancer statistics review;http://seer.cancer.gov/statfacts/html/thyro.html) with 64,300 estimated cases and 1,980 deaths in 2016 in the United States. Based on case-control studies PTC has one of the highest family risk ratios of all cancers [1, 2, 3]. Despite this, no mutations have been implicated to account for more than a tiny fraction of all PTC (see e.g. [4] for a review).

Micro RNAs (miRNAs) are an abundant class of short, noncoding RNAs that are widely expressed in mammalian cells and regulate the translation of protein-coding genes by binding to miR-specific sequences in the 3’ untranslated regions. This binding may lead to the degradation of the mRNA thus further downregulating gene expression. Strong overexpression of several miRNAs as well as modest downregulation of other miRNAs in PTC tumor tissue has been reported [5].

Sequence variants in microRNA genes were described to impact miRNA expression and function leading to human disease (for review see [6]). Most miRNAs are initially transcribed from the genome as long primary transcripts (pri-miRNAs), which are processed into precursor miRNAs (pre-miRNAs), and later into mature miRNAs. While it is known that sequence variants in the seed regions of miRNAs can affect binding of the miRNA to the target gene, it has also been shown that variants in the pre-miRNA sequence can affect the processing of the mature miRNA [7, 8]. The resulting change in the expression levels of miRNAs causes deregulation of protein-coding genes. As an example, a SNP in miR-146a predisposes to PTC via downregulation of the THRB gene [9].

We reasoned that there might be germline variants in miRNAs that affect the expression of thyroid target genes [10]. In an effort to find miRNA variants that could thus cause PTC we sequenced the genomic regions surrounding 427 selected miRs in PTC patients with a family history of the disease.

## RESULTS

### Identification of miRNA variants in PTC patients

DNA from 80 individuals affected with papillary thyroid cancer (PTC) from 80 families was subjected to SOLiD sequencing. The sequencing targeted 321 genomic regions that encode 427 miRNAs expressed in thyroid [11]. The GATK variant discovery pipeline was used according to best practices ( https://www.broadinstitute.org/gatk/guide/best-practices.php) and the short reads were aligned to hg19. We identified 1978 variants with a frequency of < 1% in 1000 Genomes Phase 1 database. To narrow down the number of variants subjected to further tests, only those (*n* = 195) that did not occur in the dbSNP database (dbSNP build 138) were considered (variants not contained in dbSNP are rare). After eliminating variants located outside of the pre-miRNA (*n* = 170), only 25 candidates were left.

### Sanger sequencing and cosegregation analysis

We then proceeded with Sanger sequencing of DNA from the individuals used for SOLiD sequencing in order to confirm the variants. Out of the 25 variants, only one in mir-450a-1 was not confirmed (Table 1).

**Table 1:** miRNA variants found in PTC families

Var	Families (#)	miRNA	Chromosomal position	Ref	Var	dbSNP	MAF (%) (1K/ESP)^b^	position (in pre-miR)	Cosegr. Analysis^c^
**1**	2	miR-200b	chr1:1102563	G	A	rs72563729	1.9/2.0	stem, not in mature mir	no
**2**	1	miR-2355	chr2:207974775	T	C	NA	NA	middle 5p	no
**3**	1	miR-15b	chr3:160122421	A	G	rs146020563	0/0.04	loop	**yes**
**4**	1	miR-15b	chr3:160122458	A	G	rs369598613	NA/0.1	stem, not in mature mir	NA
**5**	1	miR138-1	chr3:44155749	T	C	rs374104338	0.1/0.2	loop, next to 3’end of 5p	no
**6**	1	miR-135a-1	chr3:52328253	C	A	rs201615303	NA/0	3’ end of 3p	**yes**
**7**	1	miR-449a	chr5:54466362	T	C	rs144949536	0.7/0.3	stem, not in mature mir	no
**8**	1	miR-4286	chr8:10524525	G	C	rs566317227	0.1/NA	loop	NA
**9**	1	miR-320a	chr8:22102519	G	A	rs200301891	0.2/0.3	loop	**yes**
**10**	1	miR-181b-2	chr9:127456023	G	-	NA	NA	3’ end of 5p	**yes**
**11**	1	miR-199b	chr9:131007001	C	A	NA	NA	stem, not in mature mir	NA
**12**	1	miR-495	chr14:101500157	C	T	rs199783139	NA/0.04	3’ end of 3p	NA
**13**	1	miR-154	chr14:101526140	C	T	rs561595554	0/NA	loop	NA
**14**	1	miR-484	chr16:15737177	G	A	rs376603273	0.1/0.1	3’ end of 5p	**yes**
**15**	1	miR-328	chr16:67236292	C	G	rs188892061	NA/0.7	5’ end of 5p	NA
**16**	1	miR-152	chr17:46114572	C	G	rs200114569	0.6/0.4	loop	NA
**17**	2	miR-187	chr18:33484792	G	A	rs41274312	1.4/1.3	stem, not in mature mir	no
**18**	5	miR-27a	chr19:13947296	G	A	rs11671784	2.4/2.1	loop of pre-mir	no
**19**	1	miR-181d	chr19:13985711	C	T	rs367686185	NA	stem, not in mature mir	no
**20**	1	let-7e	chr19:52196093	A	G	NA	NA	5’ end of 3p	**yes**
**21**	1	miR-499	chr20:33578202	G	A	rs140486571	0.3/0.7	stem, not in mature mir	no
**22**	2	miR-296	chr20:57392686	G	A	rs117258475	1.2/1.5	3’ end of 3p	no
**23**	2	miR-130b	chr22:22007634	G	T	rs72631822	0/0	loop of pre-mir	no
**24**	1	miR-502	chrX:49779214	C	G	rs781862846	0/NA	stem, not in mature mir	NA
**25**	1	miR-450a-1	chrX:133674446^a^	C	T	NA	NA	stem, not in mature mir	NA

^a^Not confirmed by Sanger sequencing.

^b^Minor allele frequency (MAF) as reported by 1K (1000 Genomes Project phase 3) genotyping data in European population and ESP (NHLBI GO Exome Sequencing Project) in European American population.

^c^Cosegregation analysis performed (yes, variant cosegregates; no, variant does not segregate with PTC).

NA Data not available.

All variants chosen by us in the initial filtering had minor allele frequencies of less than 1% in 1000 Genomes (Phase 1 database). Later we found that of 24 variants chosen, 20 occurred in 1000 Genomes phase 3 of the database (Table 1) with frequencies up to 2.4%.

We selected 16 variants mostly for being present in a family with at least three affected individuals or for being present in more than one family. We tested them for co-segregation in all available individuals for each family which carried the trait (Supplementary Table S1).

Among the 16 tested variants, 6 showed co-segregation with the disease (namely miR-320a, miR-484, mir-15b, mir-135a, mir-181b, and let-7e; see Supplementary Table S1). Families carrying variants in miR-320a and let-7e had only two affected members but let-7e presented a high number of individuals with other types of cancers. The family carrying the variant in miR-484 was small and the inheritance pattern was not informative. We decided to use the last four miRNAs in further testing (see family pedigrees in Supplementary Figure S2). For the position of the variants in pre-miRNA see Supplementary Figure S1.

### Testing miRNA expression in patient RNA

To focus on the four miRNAs that showed co-segregation, we requested paraffin blocks of thyroid for all available individuals carrying any of the 4 variants. Unfortunately, only samples carrying miR-135a and miR-181b variants were available. Both variants were shown to be heterozygous in tumor tissue as well as in normal thyroid.

Using TaqMan assay, we tested expression of the two miRNAs (miR-135a and miR-181b) in normal and tumor tissue of both available samples (Table 2) in order to have each sample work as internal expression control for the micro RNA whose mutation is not present in the sample. The results show greatly reduced expression of miR-135a in the non-tumorous thyroid tissue from the patient with the genomic variant inside miR-135a and correspondingly repressed expression of miR-181b in tissue from the patient with a genomic variant in miR-181b. This result prompted us to test the effects of the four variant miRNAs after transfection of cell lines.

**Table 2:** TaqMan assay results of miRNA expression in tumor (T) and normal (N) thyroid tissue from 2 patients

		TaqMan Assay			
SAMPLE		miR-135a		miR-181b	
		(-dCt)	2^^^(-ddCt)	(-dCt)	2^^^(-ddCt)
miR-135a-1	T	2.70	3.51	1.53	1.41
variant carrier	N	**0.89**		**1.03**	
miR-181b-2	T	2.07	1.17	0.87	1.88
variant carrier	N	**1.84**		**–0.04**	

### Testing the effect of the variants on miRNA maturation

Since the human miR-135a is expressed from 2 genomic regions (miR-135a-1 in chromosome 3 and miR-135a-2 in chromosome 12), we tested for the expression of primary transcript of the two miRs (pri-miRNAs) in four thyroid tissue tumor/normal pairs in order to see if both pri-miRs are expressed. Both primary transcripts were expressed in the tested samples (Supplementary Figure S3A).

The same was done for miR-181b that also is expressed from 2 genomic regions (miR-181b-1 in chromosome 1 and miR-181b-2 in chromosome 9). For this microRNA too, the pri-miRNA is expressed from both genomic regions (Supplementary Figure S3B).

In order to test the effect of the variants on processing, we cloned a region of about 500 bp surrounding the pre-miRNA into an expression vector (see Materials and Methods). Constructs containing no insert (empty vector, EV), wild-type genomic region (WT), and genomic region with the variant (MUT) were transfected into the HEK293 cell line and the expression of mature miRNAs was assessed using TaqMan assays. Transfection efficiency of the plasmids in this cell line were greater than 50%. The HEK293 cell line displayed a baseline expression of all four micro RNAs (miR-15b, miR-135a, miR-181b, and let-7e) indicated by C_T_ values around 20 for all miRNAs.

**Figure 1:**
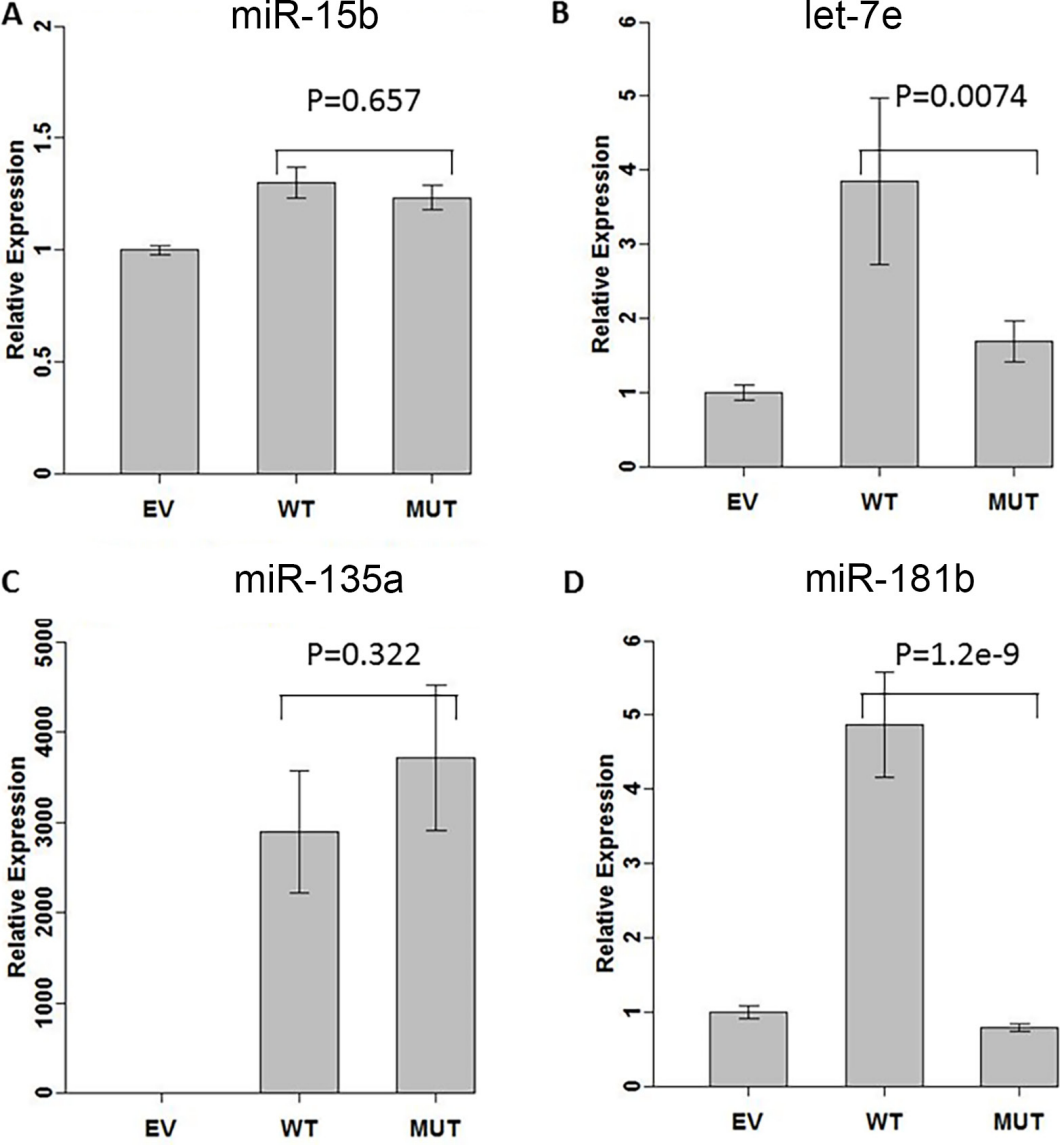
Effect of variants on miRNA expressions We measured miRNA expression of 4 miRNAs after transcfecting miRNA expressing construct without (WT) or with (MUT) the variant into HEK-293 cell line. Expression of miRNAs was normalized using basal level when transfecting with empty vector (EV). (**A**) miR-15b. (**B**) let-7e. (**C**) miR-135a. (**D**) miR-181b.

When wild-type construct was transfected, we noted a significant increase in expression for all 4 miRNAs (Figure 1). The difference in relative expression between empty vector and wild type construct is due to the amount of baseline endogenous expression of each studied miR and the amount of mature miR expressed by the plasmid.

When the mutated constructs were transfected into cell lines, there was no difference relative to WT for miR-15b and miR-135a (Figure 1A and 1B) while a significantly lower expression was measured for let-7e and miR-181b (Figure 1C and 1D).

We proceeded to test if the effect of the variants on miR maturation was similar in different cell lines. We transfected the constructs into the BCPAP and COS-7 cell lines and measured the expression of mature miR in order to see the effect of the variants on RNA processing. COS-7 was selected because of its ease of transfection and was thought to be a good cell line to test the basic differences in processing of wild-type constructs vs. mutation carriers.

The variant in the loop of pre-miR-15b showed no effect on processing of mature miRNA in either the BCPAP or the COS-7 cell lines (Supplementary Figure S4), while the variant in let-7e affected processing when the construct was transfected into the BCPAP or COS-7 cell lines (Supplementary Figure S5). The variant in miR-181b affected expression of mature miR-181b in the HEK293 (Figure 1D) and the COS-7 cell lines, but not in BCPAP (Supplementary Figure S6). The variant in miR-135a did not affect expression of the micro RNA in BCPAP or COS-7 cell lines (Supplementary Figure S7).

### Genotyping the candidate variants in PTC case/control populations

The two variants that we show to be affecting the maturation of miRNA (namely let-7e and miR-181b) are not listed in any available database (i.e. have not been seen previously) and were only found in one out of 80 families studied in our SOLiD sequencing experiment. After observing the effect of the variants on miRNA processing, we genotyped the two variants in available familial cases, sporadic cases and controls, all from Ohio. The two variants did not occur in any of the 124 familial cases tested, nor in 1170 sporadic cases or 1404 controls.

## DISCUSSION

As briefly mentioned in the Introduction, the genetic basis of PTC's high familiality is not well understood. While ionizing radiation is a well-known risk factor for the development of PTC, especially in children, it is not inconceivable that unknown environmental factors are responsible for the familial aggregation of some PTCs. Searching for hitherto unrecognized genetic and environmental factors therefore is a major challenge. In this study only heritable genetic factors are considered. Although it is becoming clear that genetic risk factors for PTC are likely to be variants with high frequency and low penetrance [12, 13, 14], here we studied familial PTC that can be expected to display low frequency and high penetrance.

Our study detected only 2 novel variants that affect miRNA expression, and both are rare, occurring in just one family each out of a total of 124 families tested. They also did not occur at all in sporadic PTC (*n* = 1170) or unaffected controls (*n* = 1404). This would appear to suggest that pathogenic genomic variants in pre-miRNA sequences are very rare, but it is worth considering the very real possibility that other variants in the same miRNAs might occur with appreciable frequency; on initial screen the SOLiD sequencing only tested 80 cases. Moreover our filtering criteria may have led us to miss some positive variants. In particular, of the 24 verified variants with the desired genomic location, only 4 remained after testing for co-segregation with disease in the families. Full co-segregation (all affected but no unaffected family members having the variant) is expected in high-penetrance genetics but may not occur with low-penetrance variants. Our conclusion that high penetrance causative variants in miR sequences are rare or very rare must be confirmed by further work.

Of the four candidate variants, the two (in miR-15b and miR-135a) in which the presence of the mutation (variant) did not alter the miRNA's expression have also been studied by others, but not implicated in thyroid cancer. The other two candidate miRNAs (let-7e and miR-181b), harboring variants that altered the abundance of their transcript, have already been well characterized in a variety of circumstances. Both are known to be implicated in thyroid cancer [15]. Previously, a higher concentration of let-7e in serum was found to be associated with PTC cases compared to controls [16].

A study published in 2014 found down-regulation of miR-181b and noted a link between microRNA down-regulation and apoptosis through targeting the CYLD gene, however, the study was done in a human papillary thyroid cancer cell line which may not adequately represent the *in vivo* situation [17]. We not that a micro RNA binding site prediction tool such as Diana Tools (http://diana.imis.athena-innovation.gr/DianaTools/) lists a large number of predicted targets for every micro RNA. This could well lead to different genes being regulated in different tissues/cell lines.

A recent review by Svoronos et al. [18] addresses the inconsistency in reports of various miRNAs being oncogenic or tumor suppressive. The authors conclude that a miRNA can act as an oncomiR or a tumor suppressor depending on the context. We conclude that the two variants found in this study are likely to be pathogenic, causing strong decrease in the expression of the two miRNAs in normal thyroid. Hypothetically, this could lead to the overexpression of a coding gene that might be an oncogene. In our study we see upregulation of miRs in tumor relative to unaffected tissue from the same organ. This is a common phenomenon [5] and could represent a defense mechanism against carcinogenesis events even in the presence of a pathogenic sequence variant in the pre-miR.

The logical follow-up of this study is searching for target genes that could act as oncogenes and definitive proof will require further experiments.

## MATERIALS AND METHODS

### Ethics statement

The experimental protocols were approved by the Institutional Review Board at the Ohio State University, and all subjects gave written informed consent before participation. Experiments were carried out in accordance with the approved guidelines.

### Individuals and clinical samples

From a total of ~180 families of PTC in our repository, we chose to study 80 families displaying Mendelian-like inheritance in at least two generations. We defined “familial” as the occurrence of at least two first or second degree relatives with PTC. Family history information, pathology reports confirming the diagnosis of thyroid cancer or other thyroid disease, and blood samples were collected from all consenting affected individuals and key unaffected individuals.

To evaluate candidate predisposing genetic factors found by the next generation sequencing (NGS), we Sanger sequenced blood DNA from an additional 44 familial cases, and genotyped 1170 Ohio sporadic cases and 1404 Ohio controls. The Ohio sporadic cases (*n* = 1170) were individuals with PTC enrolled in the Ohio State University Wexner Medical Center's (OSUWMC) endocrine neoplasia repository. Individuals were recruited from a multi-disciplinary thyroid tumor clinic at OSUWMC and all cases were histologically confirmed as PTC. Ohio control samples (*n* = 1404), matched to cases by age, gender and race were provided by the OSUWMC's Human Genetics Sample Bank. Recruitment took place in OSUWMC primary care and internal medicine clinics. All cases and controls provided written informed consent, completed a questionnaire that included demographic, medical and family history information, and donated a blood sample. Relevant clinicpathological data for cases were extracted from the electronic medical record.

### DNA and RNA extraction

Genomic DNA from blood samples was extracted using the phenol-chloroform method and stored at 4°C. DNA concentration was assessed using NanoDrop 2.0 (ThermoScientific). DNA quality was assessed by loading samples on 1% agarose gel.

Total RNA from cell lines or fresh frozen thyroid tissue was extracted by using TRIzol reagent following the manufacturer's procedure and stored at –80°C. Total RNA from paraffin block cores was extracted using NucleoSpin totalRNA FFPE extraction kit (Clontech). RNA concentration was assessed using NanoDrop 2.0 (ThermoScientific). RNA quality was analyzed with an Agilent 2100 Bioanalyzer.

### Next-generation sequencing

An Agilent SureSelect Target Enrichment kit custom designed for the 321 selected genomic regions (with mature microRNA centered and 500 bp flanking on both sides) was used. After enrichment DNAs were sonicated, gel fractionated, and the small DNA fraction (with length not exceeding 40 bp) was subjected to hybridization and ligation with Adaptor Mix. Subsequently, the DNAs were sequenced on the SOLiD platform.

### Sanger sequencing

To confirm the presence of miRNA variants and their co-segregation with PTC blood genomic DNA from patients and their available family members was Sanger sequenced. PCR primer sequences are shown in Supplementary Table S2. PCR was performed according to a standard protocol using AmpliTaq Gold DNA polymerase (Life Technologies) as follows: 10 min at 94°C; followed by 32 cycles of 15 sec at 94°C, 15 sec at 58°C, and 1 min at 72°C; followed by a final extension of 5 min at 72°C. The PCR amplicons were sequenced using an ABI3730 DNA sequencer (Genomics Shared Resource, OSU).

### Cloning of pre-miRNA regions

Pre-miRNA expression vectors were constructed by amplifying a ~0.5-kb DNA fragment encompassing the pre-miRNA region using the human genomic DNA (heterozygous for the variant of interest) as a template. Using PfuUltra high-fidelity DNA polymerase (Agilent, Santa Clara, CA, USA), PCR reactions were performed with the designed primers (Supplementary Table S2) by an Applied Biosystems 9700 Thermal cycler with annealing and elongation temperatures of 58°C and 72°C. The amplified fragments were purified with PCR purification kit (Qiagen) and analyzed on 1.5% agarose gels. The resulting fragments were cloned into a lentiviral vector (pCDH-CMV-EF1-Puro-GFP) using *Xba*I and *Bam*HI or *Nhe*I and *Bam*HI restriction enzymes (Supplementary Table S2). The plasmids that contained the wild-type or mutant miRNA genes were identified by Sanger sequencing.

### Cell lines

HEK293 and COS-7 cells were cultured in DMEM medium (Gibco) with 10% fetal bovine serum (Gibco) at 37°C in 5% CO_2_. BCPAP cells were cultured in RPMI medium (Gibco) with 10% fetal bovine serum (Gibco) also at 37°C in 5% CO_2_.

### Transient transfection

HEK293 and COS-7 cells were seeded onto 12-well plates (Corning) and transfected at 80% cell confluence with 600 ng of empty vector or vector containing wild-type or mutant insert mixed with Lipofectamine 2000 (Invitrogen; DNA to Lipo at 1:5 ratio). BCPAP cells were seeded onto 12-well plates (Corning) and transfected at 80% cell confluence with 500 ng empty vector (as control) or vector carrying wild-type or mutant miRNA region with a 1:5 ratio of DNA to Lipofectamine 2000 (Invitrogen). Transfections were viewed under a fluorescence microscope (Zeiss) and transfections were confirmed to be at least 50% efficient for HEK293 and COS-7 and ~40% efficient for BCPAP.

### Quantitative RT-PCR analysis

To quantify the expression of pri-miRNAs we used one μg of RNA in a reverse transcription reaction using the High Capacity cDNA Reverse Transcription kit including Random Hexamers (Applied Biosystems). The following TaqMan assays were used: miR-135a-1 (assay ID Hs03303129_pri), miR-135a-2 (assay ID Hs03303133_pri), miR-181b-1 (assay ID Hs03302963_pri), and miR-181b-2 (assay ID Hs03303356_pri). GAPDH was used as internal control.

To measure miRNAs expression, cDNA synthesis was performed as described by the manufacturer using TaqMan microRNA reverse transcription kit (Applied Biosystems) with the specific RT primer provided in each TaqMan assay (Applied Biosystems). The following TaqMan assays were used: miR-135a (Cat # 000460), miR-15b (Cat # 000390), miR-181b (Cat # 001098), and let-7e (Cat # 002406). RNU6B was used as internal control (Cat # 001093).

The real time PCR reactions were run on the ABI Prism 7900HT Sequence Detection System (Applied Biosystems). Fast TaqMan assay reaction mix was used and the conditions were as follows: 95°C for 5 min followed by 40 cycles at 95°C, 5 sec and 60°C, 5 sec.

### Statistical analysis

Log transformed expression data were analyzed by applying analysis of variance (ANOVA), followed by post hoc Tukey's Honest Significant Difference (HSD) test adjusting for multiple comparisons. *P*-values < 0.05 were considered as statistically significant. Results are presented as Mean ± SEM.

## SUPPLEMENTARY MATERIALS FIGURES AND TABLES






