# Supplementary material for: Variants in microRNA genes in familial papillary thyroid carcinoma
Source: Oncotarget. 2016 Dec 23;8(4):6475–82. doi: 10.18632/oncotarget.14129 (PMC5351646; doi:10.18632/oncotarget.14129)
Supplement: Supplementary file 1 [file oncotarget-08-6475-s001.pdf]

# Variants in microRNA genes in familial papillary thyroid carcinoma

## Supplementary Materials

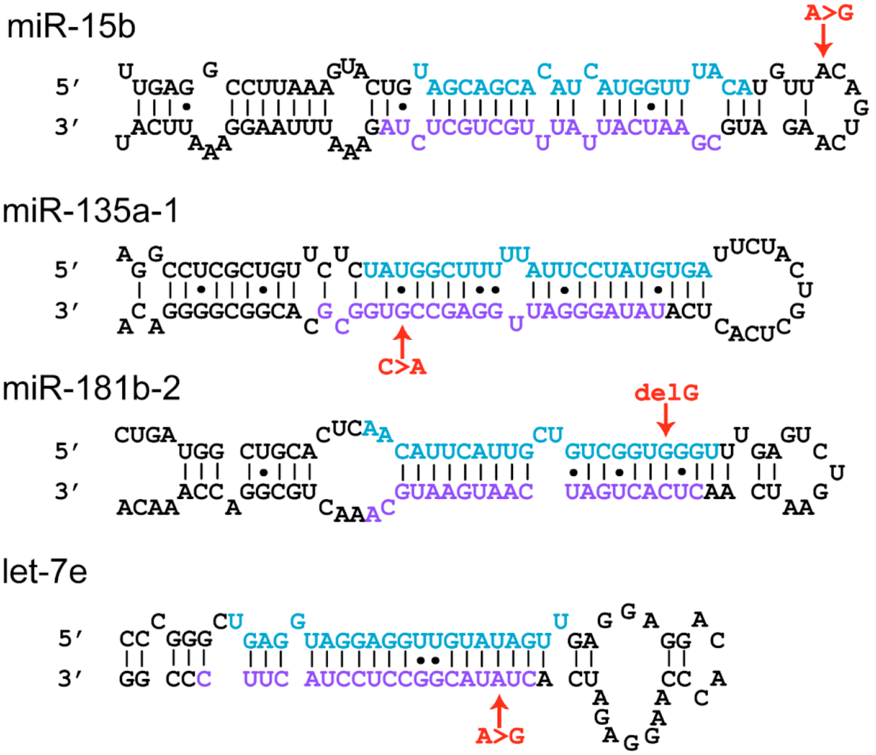


**Supplementary Figure S1: Stem-loop structure of the four miRNA precursors studied.** Red arrows indicate position of the detected variant. (structures adapted from http://mirbase.org/). Mature miRNA sequences are indicated in color.

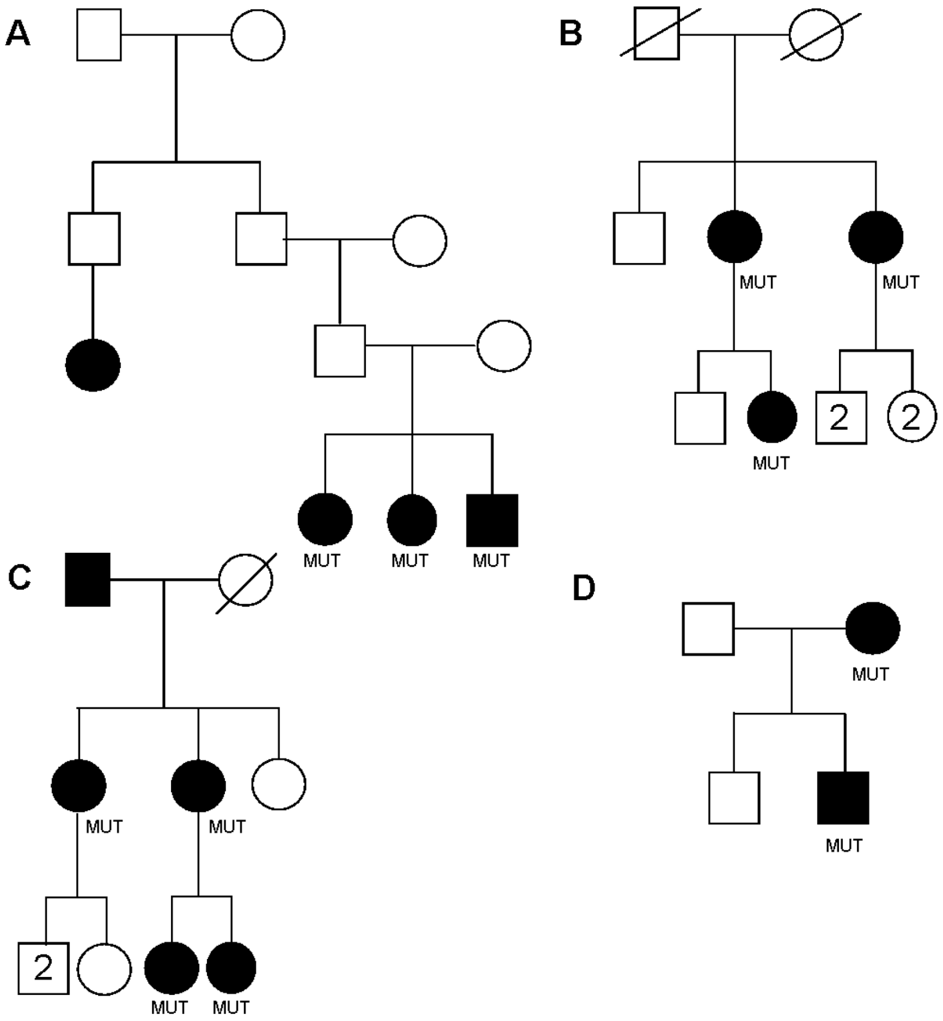


**Supplementary Figure S2: Simplified pedigrees of four families carrying the variants in 4 miRNAs tested.** (**A**) miR-15b. (**B**) miR-135a-1. (**C**) miR-181b-2. (**D**) let-7e. Circle, female; square, male; filled, PTC; MUT, heterozygous mutant.

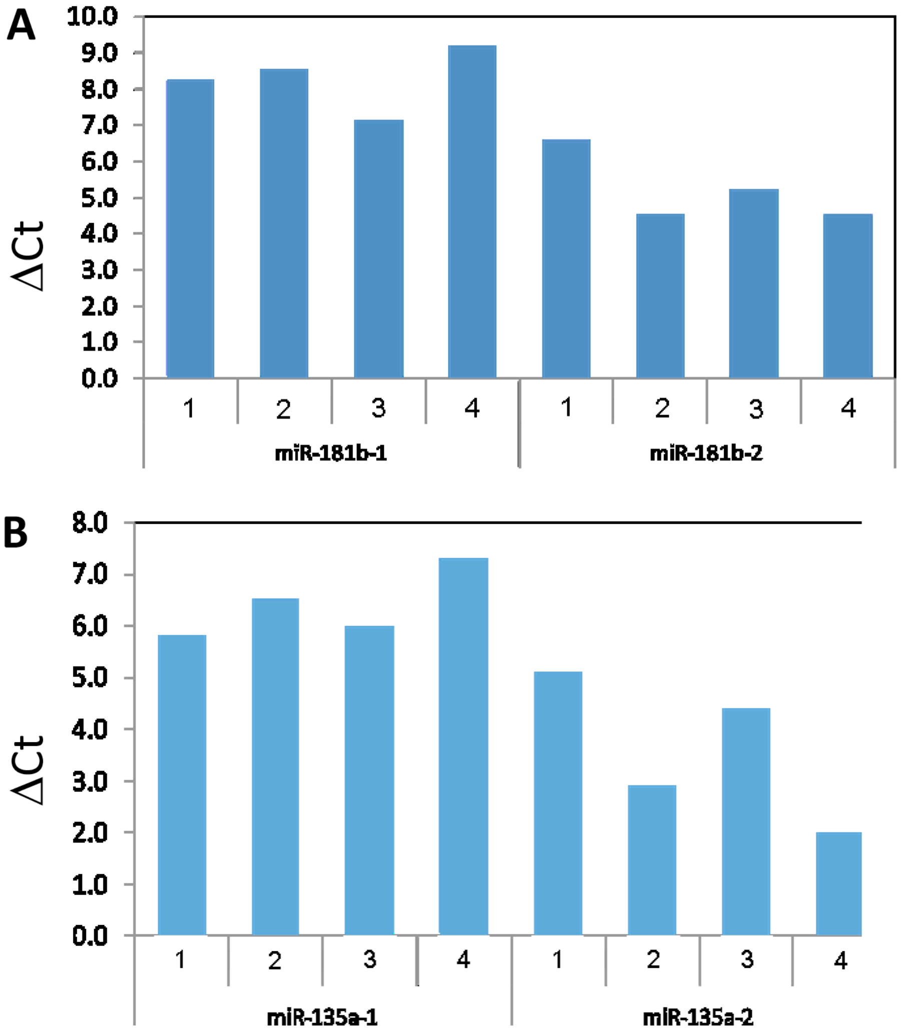


**Supplementary Figure S3: Expression of pri-miRNA in normal thyroid from 4 PTC patients.** (**A**) Expression of miR-181b-1 and miR-181b-2. (**B**) Expression of miR-135a-1 and miR-135a-2.

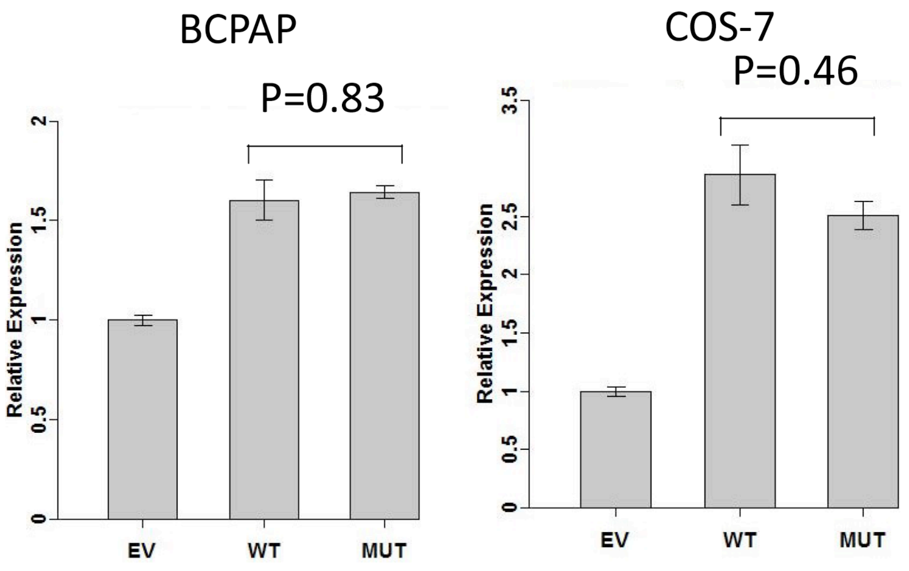


**Supplementary Figure S4: Expression of miR-15b after transfection in BCPAP and COS-7 cell lines.**

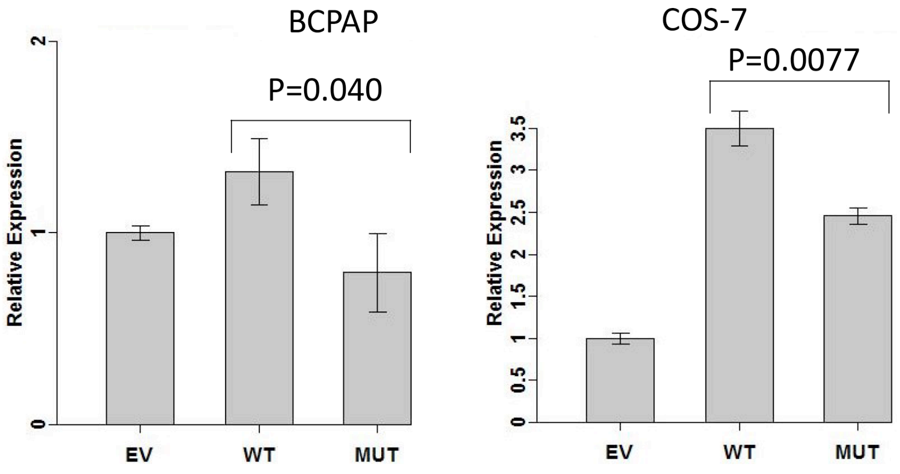


**Supplementary Figure S5: Expression of let-7e after transfection in BCPAP and COS-7 cell lines.**

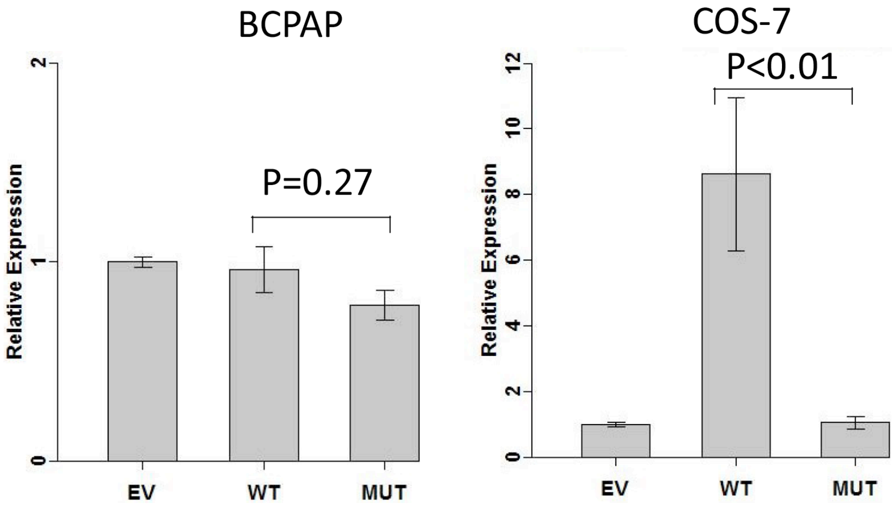


**Supplementary Figure S6: Expression of miR-181b after transfection in BCPAP and COS-7 cell lines.**

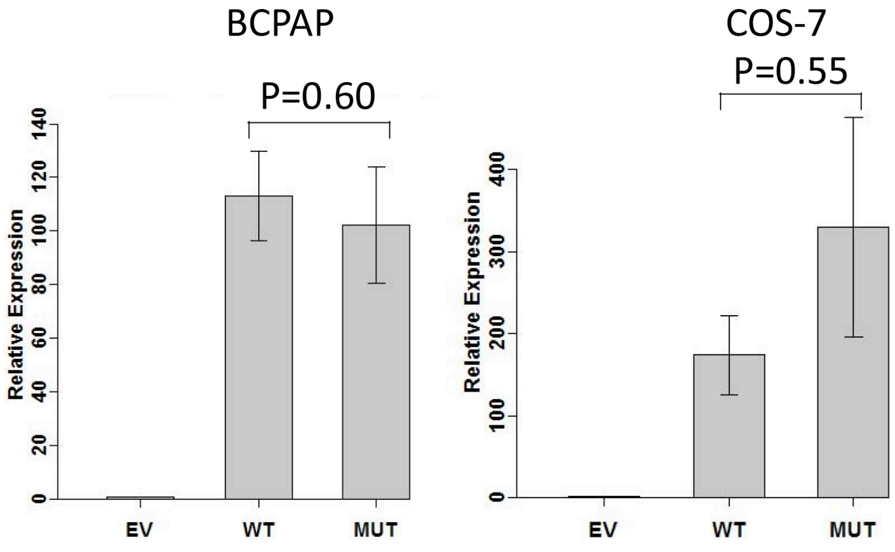


**Supplementary Figure S7: Expression of miR-135a after transfection in BCPAP and COS-7 cell lines.**

**Supplementary Table S1: Testing segregation between PTC and variant.** See Supplementary_Table_S1

**Supplementary Table S2: Primers used in the study.** See Supplementary_Table_S2
