# Supplementary material for: Variants in microRNA genes in familial papillary thyroid carcinoma
Source: Oncotarget. 2016 Dec 23;8(4):6475–82. doi: 10.18632/oncotarget.14129 (PMC5351646; doi:10.18632/oncotarget.14129)
Supplement: Supplementary file 2 [file oncotarget-08-6475-s002.docx]

| **Supplemental Table 1. Testing segregation between PTC and variant.** | | | | | |  |
| --- | --- | --- | --- | --- | --- | --- |
|  |  |  |  |  |  |  |
| **Family** | **Relationship** | **miRNA (variant position)** | **Mutation status** | **PTC status** |  |  |
| 1 | DAUGHTER | miR-200b (chr1:1102563) | Het | affected |  |  |
|  | **proband** |  | **Wild-type** | **affected** |  |  |
|  | father |  | Het | affected |  |  |
| 2 | FATHER | miR-2355 (chr2:207974775) | Het | affected |  |  |
|  | mother |  | Wild-type | Unaffected |  |  |
|  | sister |  | Het | affected |  |  |
|  | **daughter** |  | **Wild-type** | **affected** |  |  |
| 3 | BROTHER | miR-15b (chr3:160122421) | Het | affected |  |  |
|  | proband |  | Het | affected |  |  |
|  | sister |  | Het | affected |  |  |
| 4 | SISTER | mir-138-1 (chr3:44155749) | Het | affected |  |  |
|  | father |  | Het | affected |  |  |
|  | **proband** |  | **Wild-type** | **affected** |  |  |
| 5 | PROBAND | miR-135a-1 (chr3:52328253) | Het | affected |  |  |
|  | mother |  | Het | affected, fv |  |  |
|  | aunt (maternal) |  | Het | affected, fv |  |  |
| 6 | PROBAND | miR-449a (chr5:54466362) | Het | affected |  |  |
|  | sister |  | Het | affected |  |  |
|  | sister |  | Het | Goiter |  |  |
|  | **niece** |  | **Wild-type** | **affected** |  |  |
|  | niece |  | Wild-type | Goiter |  |  |
|  | niece |  | Wild-type | Goiter |  |  |
| 7 | BROTHER | miR-320a (chr8:22102519) | Het | affected |  |  |
|  | proband |  | Het | affected |  |  |
| 8 | DAUGHTER | miR-181b-2 (chr9:127456023) | Het | affected |  |  |
|  | proband |  | Het | affected |  |  |
|  | sister |  | Het | affected |  |  |
|  | daughter |  | Het | affected |  |  |
| 9 | NIECE | mir-484 (chr16:15737177) | Het | affected |  |  |
|  | proband |  | Het | affected |  |  |
|  | mother |  | Wild-type | unaffected |  |  |
|  | **sister** |  | Het | affected |  |  |
| 10 | FATHER | mir-187 (chr18:33484792) | Het | affected |  |  |
|  | **sister** |  | **Wild-type** | **affected** |  |  |
|  | mother |  | Wild-type | Unaffected |  |  |
|  | **daughter** |  | **Wild-type** | **affected** |  |  |
| 11 | NEPHEW | mir-181d (chr19:13985711) | Het | affected |  |  |
|  | **proband** |  | **Wild-type** | **affected** |  |  |
|  | **sister** |  | **Wild-type** | **affected** |  |  |
|  | daughter |  | Wild-type | Nodules |  |  |
|  | daughter |  | Wild-type | Unaffected |  |  |
|  | niece |  | Het | affected |  |  |
|  | nephew |  | Het | Unaffected |  |  |
| 12 | PROBAND | let-7e (chr19:5196093) | Het | affected |  |  |
|  | mother |  | Het | affected |  |  |
| 13 | PROBAND | mir-27a (chr19:13947296) | Het | affected |  |  |
|  | mother |  | Het | affected |  |  |
|  | **m. grandfather** |  | **Wild-type** | **affected** |  |  |
| 14 | PROBAND |  | Het | affected |  |  |
|  | **sister** |  | **Wild-type** | **affected** |  |  |
| 15 | PROBAND |  | Het | affected |  |  |
|  | **sister** |  | **Wild-type** | **affected** |  |  |
| 16 | SISTER |  | Het | affected |  |  |
|  | **sister** |  | **Wild-type** | **affected** |  |  |
| 17 | PROBAND |  | Het | affected |  |  |
|  | sister |  | Het | affected |  |  |
| 18 | SISTER | miR-296 (chr20:57392686) | Het | affected |  |  |
|  | **proband** |  | Wild-type | affected |  |  |
| 19 | SISTER | miR-499 (chr20:33578202) | Het | affected |  |  |
|  | **proband** |  | **Wild-type** | **affected** |  |  |
|  | **sister** |  | **Wild-type** | **affected** |  |  |
|  | sister |  | Het | affected |  |  |
| 20 | PROBAND | miR-130b (chr22:22007634) | Het | affected |  |  |
|  | mother |  | Het | Unaffected |  |  |
|  | father |  | Wild-type | Unaffected |  |  |
|  | **sister** |  | **Wild-type** | **affected** |  |  |
|  | **brother** |  | **Wild-type** | **affected** |  |  |
|  |  |  |  |  |  |  |
| Individuals sequenced (SOLiD) in capital letters; affected but wild-type in bold | | | | | | |
