# Supplementary material for: Variants in microRNA genes in familial papillary thyroid carcinoma
Source: Oncotarget. 2016 Dec 23;8(4):6475–82. doi: 10.18632/oncotarget.14129 (PMC5351646; doi:10.18632/oncotarget.14129)
Supplement: Supplementary file 3 [file oncotarget-08-6475-s003.docx]

| **Supplemental Table 2. Primers used in the study.** | | |
| --- | --- | --- |
|  |  |  |
| **Variant screening** | Primer | Sequence (5' to 3') |
| mir-200b | Forward Primer | CCCCAGCTACTGAGCTTC |
|  | Reverse Primer | TCACTCTCCCCAGAGCCATC |
| mir-2355 | Forward Primer | GCCCTTTCAGCTACATTCAC |
|  | Reverse Primer | TTCAAGTGGCAAGGGATAAG |
| mir-15b | Forward Primer | TCTTCGAAGCCATGGAATTG |
|  | Reverse Primer | TTGCTGTATCCCTGTCACAC |
| mir-138-1 | Forward Primer | GTTCTCACTTCTGGCTGTTC |
|  | Reverse Primer | CCAGCCCAGAACTGGAAAG |
| mir-135a-1 | Forward Primer | GGTCATGAACTGTCTGCAAG |
|  | Reverse Primer | GAAGAGGCAAGTGGGAATAG |
| mir-449a | Forward Primer | AAGTCAGCAGCCACAACTAC |
|  | Reverse Primer | GCTTGCTTCATAGCAGAAAG |
| mir-4286 | Forward Primer | AGACTGTCCCACCCTAGTTC |
|  | Reverse Primer | CTGGGAGATGTCAGCTTATG |
| mir-320a | Forward Primer | GTCACAACCTCACCTGCAAC |
|  | Reverse Primer | CGAGGCGAATCCTCACATTG |
| mir-181b-2 | Forward Primer | AAGCCAGACACACAGACTTC |
|  | Reverse Primer | CTCCCACTCACAGCAGTAG |
| mir-199b | Forward Primer | CACGTCAAAGGAGGCAGAAG |
|  | Reverse Primer | CCCCATCCTCTCAGTCTTC |
| mir-495 | Forward Primer | CTGGGGTGAGCCAACTTAC |
|  | Reverse Primer | AGGACTGAGGACTGAGCATG |
| mir-154 | Forward Primer | TCTGCTAACCGTCAATGTTC |
|  | Reverse Primer | GTGGCACCATCTCTAGAAAC |
| mir-484 | Forward Primer | ACGCCCTTCTCTCCTCTTC |
|  | Reverse Primer | AGCCTGAAAGAGCTGAATTC |
| mir-328 | Forward Primer | TCGTGGAAGCTCTGGTCTTG |
|  | Reverse Primer | CCCAGTTTGTGTTGGAGAAC |
| mir-152 | Forward Primer | ATGGGCATGCTTCTGGAGTC |
|  | Reverse Primer | CAGTCCTCAAGGTCCACAG |
| mir-187 | Forward Primer | TCGGATTCCCCAAGCAGAAG |
|  | Reverse Primer | CTGGACTTTCCCATGGTCTC |
| mir-27a | Forward Primer | CTGGGGATGGGATTTGCTTC |
|  | Reverse Primer | GCCAGTGTACACAAACCAAC |
| mir-181d | Forward Primer | TCAACCTGTCGGTGAGTTTG |
|  | Reverse Primer | TCTCCAAAGAGGAGTGGTTG |
| let-7e | Forward Primer | ACCCGTAGAACCGACCTTG |
|  | Reverse Primer | GCAGAGATGGAGACAGACAG |
| mir-499 | Forward Primer | TCCCAGCTGCACAAGGTAAG |
|  | Reverse Primer | TCACCACCACCAAAGTCTTC |
| mir-296 | Forward Primer | AGGAGGGAAGATCCTGAGTG |
|  | Reverse Primer | GTGGGAGGTAGGTAAAACTC |
| mir-130b (1) | Forward Primer | TGCATTCCAGGTCTCAGATC |
|  | Reverse Primer | TGGAAAGTGGAGTGAGGCAG |
| mir-502 | Forward Primer | CAAGACTCTCATTCCTGTTC |
|  | Reverse Primer | CCTGAAGTCCCTAACTATTC |
| mir-450a-1 | Forward Primer | GAGGCTATCAGGAAGTATAC |
|  | Reverse Primer | TGGCCAAATAGGAAGTCTTC |
|  |  |  |
| **Variant cloning** |  |  |
| let7e | XbaI fw | aacttctagaACCCGTAGAACCGACCTTG |
|  | BamHI rv | aatcggatccGCAGAGATGGAGACAGACAG |
| 181b-2 | XbaI fw | aatctctagaAAGCCAGACACACAGACTTC |
|  | BamHI rv | aatcggatccTACTCCATGTTAGAACCAAG |
| 15b | NheI fw | aatcgctagcTCTTCGAAGCCATGGAATTG |
|  | BamHI rv | aatcggatccTTGCTGTATCCCTGTCACAC |
| 135a-1 | Xba fw | aatctctagaGGTCATGAACTGTCTGCAAG |
|  | BamHI rv | aatcggatccGAAGAGGCAAGTGGGAATAG |
